# MicroRNAs modulate CaMKIIα/SIRT1 signaling pathway as a biomarker of cognitive ability in adolescents

**DOI:** 10.1016/j.bbih.2025.100970

**Published:** 2025-02-24

**Authors:** Li-Ching Lee, Ming-Tsan Su, Lei Bao, Po-Lei Lee, Shane Tutwiler, Ting-Kuang Yeh, Chun-Yen Chang

**Affiliations:** a Institute for Research Excellence in Learning Sciences and Graduate Institute of Science Education, National Taiwan Normal University, Taipei, Taiwan; b Department of Life Science, National Taiwan Normal University, Taipei, Taiwan; c Department of Physics, The Ohio State University, Columbus, OH, USA; d Department of Electrical Engineering, National Central University, Taoyuan City, Taiwan; e Learning Foundations, University of Rhode Island, Kingston, RI, USA; f Institute of Marine Environment Science and Technology, National Taiwan Normal University, Taipei, Taiwan; g Department of Earth Sciences, National Taiwan Normal University, Taipei, Taiwan; h Department of Biology, Universitas Negeri Malang, Indonesia

**Keywords:** CaMKIIα/SIRT1 signaling pathway, miRNA, NF-kB, Cognitive ability, Adolescents

## Abstract

The dynamic regulation of synaptic plasticity underlies memory formation, involving intricate signaling pathways with both facilitatory and inhibitory roles. MicroRNAs are emerging modulators of memory processes through their fine-tuning of gene expression. To explore the influence of miRNAs on adolescent cognitive function, we investigated the association between academic performance, cognitive ability as measured by the Inquiry for Scientific Thinking, Analytics, and Reasoning test, and plasma miRNA profiling in 486 senior high school students. Our analysis identified 38 differentially expressed miRNAs between students with high and low academic performance. Notably, miR-219 b/548e/628/885 and miR-30a/30c-1/195/204 potentially targeted genes associated with the CaMKII/SIRT1 signaling pathway, a crucial facilitator of memory consolidation. Collectively, our findings suggest that specific plasma miRNAs, particularly the CaMKII/SIRT1-related miR-30a/30c-1/195/204 cluster, potentially serve as promising biomarkers for cognitive function in adolescents. Our findings further support the proposed interaction between NF-kB activity and CaMKIIα in regulating synaptic plasticity. Under hypomethylation conditions, increased NF-kB activity, a key component of inflammation and neural plasticity, influences learning and memory. This biological pathway, representing the initiation of epigenetic memory, demonstrates significant predictive power for both cognitive ability and academic performance.

## 1 Introduction

Over the past two decades, epigenetic regulation mechanisms, such as dynamic DNA methylation reactions and chromatin remodeling, have been identified as integral processes in long-term memory formation, maintenance, and recall (Kennedy and Sweatt, 2016; Kim and Kaang, 2017). The brain's remarkable plasticity enables it to adapt to environmental changes. Epigenetic modifications are modifications influenced by environmental factors that contribute to the formation of epigenetic memory, a heritable change in gene expression of behavior, induced by a previous stimulus (D'Urso and Brickner, 2014). Memory occurs by multiple mechanisms but usually requires chromatin-base changes such as DNA methylation, histone modifications, or incorporating variant histones, affecting brain function throughout life. Higher brain functions, such as learning, memory, and cognition, presumably rely on long-term changes in synaptic strength, including long-term potentiation (LTP) and long-term depression (LTD). These opposing forms of synaptic plasticity play a key role in neural circuit remodeling and the formation of memory engrams. Engrams emerging as the basic unit of memory: Though scientist Richard Semon introduced the concept of the “engrams" 115 years ago to posit a neural basis for memory, direct evidence for engrams has only begun to accumulate recently as sophisticated technologies and methods have become available by recently. For instance, evidence indicates that increased intrinsic excitability and synaptic plasticity work hand in hand to form engrams. These processes may also be important in memory linking, retrieval, and consolidation by Prof. Susumu Tonegawa's research team. (Josselyn and Tonegawa, 2020; Liu et al., 2012; Redondo et al., 2014).

Calcium/calmodulin-dependent protein kinase II (CaMKII) is a key signaling molecule involved in both LTP and LTD. Recent studies have indicated that the relative phosphorylation state of CaMKII at threonine 286 (pT286) and threonine 305/306 (pT305/306) plays a crucial role in determining the direction of synaptic plasticity (Piochon et al., 2016). LTP and LTD require T286 phosphorylation, but T305/306 phosphorylation selectively promotes LTD. This differential regulation of CaMKII activity is essential for appropriate neural circuit function and the formation of stable memory traces (Cook et al., 2021, Cook et al., 2022). Overall, these findings highlight the importance of CaMKII phosphorylation dynamics in orchestrating synaptic plasticity, and they also underscore the molecular aspects of higher brain functions. Understanding the mechanisms underlying the regulation of CaMKII activity may facilitate the development of novel therapeutic strategies for neurological disorders associated with learning and memory impairments.

Gene expression is the biological process of translating coded instructions from the genome into functional molecules that affect specific cell processes. Epigenetic modifications may affect gene expression, facilitating cross-talk between genes and the environment (Turner, 2009) or clinical manifestation (Recabarren-Leiva and Alarcón, 2018; Song et al., 2022). Epigenetic regulation of a gene expression may occur through direct or indirect interactions with histones, such as histone acetylation, deacetylation, DNA methylation, and demethylation. Sirtuin 1 (SIRT1) is a histone deacetylase that promotes gene expression by targeting specific promoter regions. It has been shown that SIRT1 upregulates brain-derived neurotrophic factor (BDNF) expression by targeting its promoter region and activating the cAMP-response element-binding protein (CREB)- transducer of regulated CREB activity 1 (TORC1) signaling pathway (Jeong et al., 2011). SIRT1 overexpression is associated with reduced learning and memory impairments, suggesting a neuroprotective role of SIRT1 in cognitive function (Corpas et al., 2017). SIRT1 also plays a role in regulating the expression of CaMKIIα by repressing the transcription of CaMKIIα and deacetylate H3K9 at the CaMKIIα promoter (Zhou et al., 2020). In addition, SIRT1 exerts a neuroprotective effect through the SIRT1/miRNA-134 pathway, which regulates synaptic transcriptional proteins such as BDNF and CREB (Shen et al., 2018). In summary, SIRT1 plays a key role in regulating gene expression and epigenetic modifications, with implications for various cellular processes, including learning, memory, pain perception, and neuroprotection.

Despite the major advances made in understanding the functions of SIRT1 in cognition, the mechanisms by which it is regulated remain largely unclear. In various cognitive impairments, such as dementia, SIRT1 has aberrant functions, indicating that it plays a key role in physiological memory function and the epigenetic regulation of synaptic protein expression and that it increases the strength and efficacy of synaptic plasticity (Fagerli et al., 2022). Intriguingly, SIRT1-mediated deacetylation may be influenced by the binding of methyl-CpG binding protein 2 (MeCP2) to methylated DNA (Zocchi and Sassone-Corsi, 2012). This interaction indicates that the potential interplay between SIRT1 and MeCP2 plays a role in regulating gene expression. Given the strong connections between SIRT1 and metabolic function, it is conceivable that specific neurons' metabolic states may modulate MeCP2 and CaMKIIα. Further research into these potential connections may provide valuable insights into the complex mechanisms underlying memory formation and cognitive function.

Epigenetics is the study of chromatin alterations that modify the transcription of genes. This branch of science has recently gained popularity because it shapes long-term brain plasticity and function. Epigenetic modifications, influenced by environmental cues such as diet and stress (Reemst et al., 2024), act as molecular switches that can turn genes on or off, thereby shaping brain function throughout an individual's lifespan (Engdahl et al., 2021; Malanchini et al., 2020; Raffington et al., 2023). Cognitive ability and educational attainment are strongly linked to positive life outcomes, including high earnings, favorable health conditions, and increased longevity (Belsky et al., 2019; Dabiri et al., 2024; Schlichting et al., 2022).

Recently, microRNAs (miRNAs) have emerged as key regulators of synaptic efficacy, positively or negatively influencing memory formation (Feng et al., 2017; Peixoto et al., 2015). In addition to causing changes in gene expression, epigenetics is a mechanism through which learning experience can modify synaptic plasticity, memory acquisition, and memory consolidation. During development and throughout adulthood, genetic predisposition, experience, and neuronal activity interact to shape cellular and behavioral responses (Mellios et al., 2008; Sinha et al., 2020). Neuromodulation is regarded as a promising technique for enhancing memory function, and understanding the epigenetic mechanisms underlying this process may provide insights into the molecular aspects of cognition (Bastle et al., 2018; Poon et al., 2020). Therefore, memory-related research has focused on investigating the epigenetic mechanisms of gene expression regulation in behavior and memory propagation, revealing the central role of epigenetic regulation of gene expression in establishing genetic memory that influences long-term brain function and behavior (Barnett et al., 2023; Fernandes et al., 2017).

This study examined the epigenetic mechanisms underlying memory formation and the relationship between these mechanisms and adolescents' cognitive ability and academic performance. By examining the molecular aspects of these processes, our goal was to gain deeper insights into the biological foundations of cognitive processes and develop strategies to enhance learning and memory.

## 2 Materials and methods

### 2.1 Participants

This study recruited 486 students (286 boys and 200 girls, 16.8 ± 0.31 years of age) from senior high schools in central Taiwan (approved by the Institutional Review Board of National Taiwan Normal University—REC no. 202105HM013). The participants and their parents were explicitly informed of the study's purpose, and written consent was obtained.

The participants’ backgrounds and three feature selection methods (test evaluation) were shown as demographic characteristics (Table 1).

**Table 1:** Demographic characteristics.

	Male (n = 288)	Female (n = 198)	*P*
Age (yes)	16.8 ± 0.32^a^	16.8 ± 0.30	
Academic performance (CAP)	86.77 ± 0.73	84.26 ± 0.92	P < 0.05
Cognitive abilities (iSTAR)	8.36 ± 0.20	8.25 ± 0.22	
control of variables (COV)	3.89 ± 0.10	3.87 ± 0.11	
data analytics (DA)	2.20 ± 0.07	2.07 ± 0.09	
causal decision making (CDM)	2.26 ± 0.08	2.31 ± 0.09	
Situation-based career interest assessment (SCIA)			
scientific reasoning	112.08 ± 0.82	110.88 ± 0.87	

a Mean values (standard deviation).

### 2.2 Cognitive ability and academic performance assessments

The present study collected data on participants' academic performance, Inquiry for Scientific Thinking, Analytics, and Reasoning (iSTAR), as well as their scientific reasoning ability. For assessing academic performance, the Comprehensive Assessment Program (CAP) test of Taiwan, a standardized annual competency test was used. A performance rating higher than 85 was designated as the higher academic performance group, whereas a performance rating lower than 60 was designated as the lower academic performance group.

iSTAR was developed by the Bao research team (Bao et al., 2022). Provides a theoretical model and assessment instrument focused on understanding and evaluating students' scientific reasoning abilities, which emphasize three areas of core skills that support scientific inquiry, including control of variables (COV), data analytics (DA), and causal decision-making (CDM). The iSTAR assessment instrument used in this study is an 18-question multiple-choice test. The assessment results can provide evaluations of students’ overall proficiency in scientific reasoning as well as their performance levels in the three skill dimensions on COV, DA, and CDM.

Participants' scientific reasoning ability was assessed using the situation-based career interest assessment (SCIA) (Sung et al., 2016). This revised version of the SCIA not only predominantly evaluates the cognitive abilities or personality tendencies of test takers but also can measure the capacity to gauge career interests. It has provided a direct and tangible contribution to the development of career guidance tools customized for adolescent students facing educational streaming while attending senior high schools. The SCIA comprises eight subtests for space, language (Chinese), mathematics, scientific reasoning, logical reasoning, aesthetics, creativity, and foreign language. In this study, we focused on students' scientific reasoning capabilities which represent the cognitive abilities of Taiwanese adolescents.

### 2.3 Human plasma miRNA profiling

Blood samples were collected in tubes containing ethylenediaminetetraacetic acid (EDTA) and the plasma was obtained by centrifuging at 2000×*g* for 15 min at 4 °C, and stored at −80 °C before miRNA extraction and sequencing. MiRNA was extracted from 40 pre-screened samples using the miRNeasy Serum/Plasma Kit (Qiagen, Hilden, Germany). Small RNA libraries were prepared using Ion Total RNA-Seq Kit v2 (Life Technologies, Carlsbad, CA, USA). These libraries were pooled in an equal molar ratio for template bead preparation. Subsequently, sequencing beads were prepared and enriched in accordance with the manufacturer's instruction (Ion Chef, Next-Generation Sequencing). Sequencing was performed using a 540 chip on Ion GeneStudio S5 Prime (Thermo Fisher Scientific, Waltham, MA, USA) in accordance with the aforementioned protocol of the Ion 540 Kit-Chef (2 sequencing runs per initialization). Alignment to the hg19 human reference genome and base calling were performed using built-in Torrent Suite Software (Thermo Fisher Scientific, Waltham, MA, USA) v5.16.1. Analysis of miRNA expression was conducted using Partek Genomics Suite v7.0. The sequencing coverage of “Human plasma miRNA profiling” is shown in Supplementary Data Table S1. Differentially expressed miRNAs with p-value and up/down fold change (FC) > 3 were identified and indicated in supplementary data (Extended data Excel file 1–4 for human plasma miRNA profiling).

### 2.4 Human plasma miRNA profiling was screened (40 pre-screen samples) using different feature selection methods (test evaluation). Shown in supplementary

To investigate the potential association between microRNA (miRNA) expression and academic performance, miRNA profiling was performed on plasma samples collected from a cohort of students. A performance rating higher than 85 was designated as the higher academic performance group, whereas a performance rating lower than 60 was designated as the lower academic performance group.

Plasma miRNA expression profiles from a cohort of 40 students were compared based on the logits of the iSTAR assessment. Those iSTAR scoring ≥ 9 designated as the higher performance group (n = 20), while those scoring <9 were designated as the lower performance group (n = 20).

Plasma miRNA expression profiles from a cohort of 40 students were compared based on the scientific reasoning score in SCIA. A score higher than 108 was designated as the higher scientific reasoning group (n = 20), whereas a score lower than 108 was designated as the lower scientific reasoning group (n = 20) (Total score was 140).

### 2.5 Real-time quantitative polymerase chain reaction

Total RNA was extracted from 625 μL of human plasma by using the mirVana PARIS extraction kit (Ambion, Carlsbad, CA, USA) following the manufacturer's instruction. A total of 12.5 ng RNA were subjected to the real-time quantitative polymerase chain reactions (qPCR) on a ViiA 7 Real-Time PCR System (Life Technologies) using a TaqMan Reverse Transcription Kit (Life Technologies, Thermo Fisher Scientific) and miRNA-specific stem-loop primers (Applied Biosystems, Waltham, MA, USA). Primer sets for mature miRNAs were designed using Applied Biosystems TaqMan Small RNA Assays RNU6B(Assay ID 001093), has-miR-30a-5p (Assay ID 000417), has-miR-30c-1-3p (Assay ID 002108), has-miR-195–5p (Assay ID 000494), has-miR-204–5p (Assay ID 000508) and the following TaqMan probes were used for gene expression analysis: MeCP2 (Hs00172845_m1), SIRT1 (Hs01009006_m1), HDAC9 (Hs01081558), BDNF (Hs02718934_s1), and hypoxanthine-guanine phosphoribosyltransferase (HPRT, Hs99999909_m1, Applied Biosystems), which served as the endogenous control. Fold changes were calculated using the 2^ΔCt^ method, ΔC_T_ = C_T_ (HPRT1) − C_T_ (target gene), where C_T_ indicates the cycle threshold (Cosentino et al., 2022).

### 2.6 Enzyme-linked immunosorbent assay

The blood plasma, obtained as mentioned above, was centrifuged for 10 min at 10,000×*g* to remove platelets. To quantify the levels of CaMKIIα, 100 μL of plasma and an enzyme-linked immunosorbent assay (ELISA) kit were used following the manufacturer's instructions (Cat. No. LS-F20920, LifeSpan BioSciences, Shirley, WA, USA).

### 2.7 Cell culture and transfection

HEK293 cells expressing either CaMKIIα or MeCP2 was generated using an inducible Flp-In T-REx system (Invitrogen, Carlsbad, CA, USA). Briefly, the HEK293 cells were cotransfected with a pOG 44 plasmid (constitutively expressing Flp recombinase) and a pcDNA5/FRT/TO-MeCP2 plasmid, and the stably transfected cells were obtained as per the supplier's instructions. HEK 293-derived Flp-In host cells, expressing either CaMKIIα or MeCP2, were generated as previously described (Lee et al., 2012). Cell lines were cultured will be shown in the supplementary text.

### 2.8 Western blotting

Total soluble protein was obtained by centrifugation at 15,000×*g* for 10 min at 4 °C. Protein concentrations were determined using the Bradford assay (Cat. No. 5000002; Bio-Rad Laboratories Hercules, CA, USA). For each sample, a total of 25 μg proteins were separated using 12% sodium dodecyl sulfate–polyacrylamide gel electrophoresis (SDS-PAGE), and electroblotted onto nitrocellulose membranes. Details about the western blotting process will be shown in the supplementary text.

### 2.9 Statistical analysis

For each set of values, data are expressed as mean ± standard deviation (SD) of three independent experiments. Differences between groups were analyzed using Student's *t*-test or one-way or two-way analysis of variance (ANOVA) supplementary data Table S5, with a post hoc least significant difference test where appropriate. A *P* value less than 0.05 was considered statistically significant. Tests of assumptions for ANOVA and inferential statistical analyses were conducted using IBM SPSS Statistics v.23.0 (IBM, Armonk, NY, USA).

## 3 Results

### 3.1 Academic performance (CAP), cognitive abilities (iSTAR), scientific reasoning in (SCIA) test assessment, and human plasma samples

In the present study, 486 students (286 boys and 200 girls, 16.8 ± 0.31 years of age) volunteers were recruited for the study. Students’ performance tests were assessed in different gender (Table 2). Prof. Bao's research team developed a comprehensive model of scientific reasoning and a valid assessment tool called iSTAR to target the capabilities of scientific reasoning and critical thinking, among the diverse skills required for 21st-century learners. Pearson's correlation analysis revealed a correlation between academic performance (CAP) and cognitive ability (iSTAR), indicating a positive relationship between the two measures (Supplementary Table 2).

**Table 2:** Students’ performance tests assessment in different gender.

	Male (n = 40)	Female (n = 38)	*P*
Academic performance (CAP)	87.43^a^ ± 3.01	86.68 ± 3.44	
Cognitive abilities (iSTAR)	8.23 ± 0.79	7.95 ± 0.49	
control of variables (COV)	3.98 ± 0.23	3.61 ± 0.26	
data analytics (DA)	1.93 ± 0.29	1.87 ± 0.20	
causal decision making (CDM)	2.33 ± 0.22	2.47 ± 0.21	
Situation-based career interest assessment (SCIA)			
scientific reasoning	113.65 ± 2.32	114.24 ± 2.03	

a Mean values (standard deviation).

From the results of Table 3, Table 4, the plasma CaMKIIα levels (ELISA, pg/mL) were positively and significantly linked with academic performance (CAP) and cognitive ability (iSTAR), and the plasma SIRT1 was negatively and significantly linked with cognitive ability (iSTAR). In Table 3 Differences in plasma biomarkers between high and low achievers on iSTAR, including its three skill dimensions: variable control, data analysis, and decision-making. Only variable control showed no linked with SIRT1 mRNA gene expression level.

**Table 3:** Differences in plasma biomarkers (gene expression levels) between high and low achievers on academic performance (CAP) and cognitive ability (iSTAR).

	MeCP2 mRNA		BDNF mRNA		SIRT1 mRNA		HDAC9 mRNA		ELISA CaMKIIα	
**CAP score**										
**(n = 39) CAP score** ≥ **83**	5.28 (5.45)^a^	*T = 1.495*	1.98 (1.77)	*T = 0.558*	0.31 (0.18)	*T = -2.883*	2.73 (1.62)	*T = 0.841*	536.4 (243.9)	*T = 3.700*
**(n = 39) CAP score < 83**	3.74 (3.40)	*P = 0.140*	1.76 (1.77)	*P = 0.578*	0.41 (0.29)	*P = 0.773*	2.33 (1.40)	*P* = *0.405*	363.2 (161.2)	***P = 0.000***

**iSTAR test**										
**(n = 36) iSTAR score** ≥ **9**	5.05 (5.22)	*T = 0.952*	1.98 (1.79)	*T = 0.513*	0.27 (0.16)	*T = -2.833*	1.99 (1.61)	*T = 0.016*	521.6 (269.1)	*T = 2.74*
**(n = 42) iSTAR score < 9**	4.04 (3.94)	*P = 0.334*	2.53 (2.08)	*P = 0.610*	0.42 (0.28)	***P = 0.006***	1.99 (1.48)	*P = 0.988*	388.3 (152.5)	***P = 0.008***

- **Control of variable**										
**(n = 29) score** ≥ **5**	5.09 (5.58)	*T = 0.788*	1.97 (1.92)	*T = 0.372*	0.31 (0.20)	*T = -1.454*	2.20 (1.85)	*T = 0.855*	541.6 (290.3)	*T = 2.929*
**(n = 49) score < 5**	4.16 (3.88)	*P = 0.435*	1.81 (1.67)	*P = 0.711*	0.38 (0.27)	*P = 0.150*	1.87 (1.32)	*P = 0.397*	395.5 (150.2)	***P = 0.004***

- **Data analysis**										
**(n = 48) score** ≥ **2**	4.38 (4.94)	*T = -0.319*	1.87 (1.72)	*T = 0.044*	0.31 (0.21)	*T = -2.093*	1.83 (1.51)	*T = -1.141*	491.2 (249.8)	*T = 2.120*
**(n = 30) score < 2**	4.71 (4.00)	*P = 0.751*	1.86 (1.85)	*P = 0.965*	0.44 (0.32)	***P = 0.043***	2.24 (1.56)	*P = 0.258*	383.6 (153.8)	***P = 0.037***

- **Causal decision-making**										
**(n = 35) score** ≥ **3**	4.34 (4.03)	*T = -0.298*	1.91 (1.77)	*T = 0.177*	0.30 (0.17)	*T = -2.192*	1.85 (1.41)	*T = -0.729*	514.3 (271.6)	*T = 2.371*
**(n = 43) score < 3**	4.64 (5.02)	*P = 0.766*	1.84 (1.77)	*P = 0.860*	0.41 (0.29)	***P = 0.032***	2.10 (1.63)	*P = 0.468*	397.3 (158.9)	***P = 0.020***

**Gender different Group**										
**male = 40**	4.65 (4.17)	*T = 0.277*	1.87 (1.77)	*T = 0.013*	0.42 (0.30)	*T = 2.647*	2.26 (1.57)	*T = 0.841*	469.6 (271.3)	*T = 0.804*
**female = 40**	4.36 (5.02)	*P = 0.782*	1.86 (1.77)	*P = 0.990*	0.29 (0.15)	***P = 0.010***	1.71 (1.46)	*P = 0.405*	428.9 (158.5)	*P = 0.424*

a Mean (SD).

**Table 4:** Differences in plasma biomarkers (miRNA expression level) between high and low achievers on academic performance (CAP) and cognitive ability (iSTAR).

	miR-30a-5p		miR-30c-1-3p		miR-195–5p		miR-204–5p	
**Comprehensive Assessment Program**								
**(n = 39) CAP score** ≥ **83**	43.25 (74.17)^a^	*T = 1.246*	1.01 (0.55)	*T = 2.345*	0.80 (0.98)	*T = -0.575*	1.34 (1.64)	*T = -0.419*
**(n = 39) CAP score < 83**	26.22 (42.28)	*P = 0.218*	0.71 (0.58)	***P = 0.022***	0.91 (0.75)	*P = 0.567*	1.71 (5.35)	*P* = 0.677

**Science inquiry ability iSTAR test**								
**(n = 36) iSTAR score** ≥ **9**	41.89 (72.23)	*T = 0.965*	0.89 (0.51)	*T = 0.364*	0.82 (1.02)	*T = -0.362*	1.17 (1.44)	*T = -0.76*
**(n = 42) iSTAR score < 9**	28.60 (48.54)	*P = 0.337*	0.84 (0.64)	*P = 0.717*	0.89 (0.73)	*P = 0.718*	1.84 (5.20)	*P = 0.451*

**Gender different Group**								
**male = 40**	35.79 (58.53)	*T = 0.157*	0.92 (0.66)	*T = 0.935*	0.76 (0.51)	*T = -1.031*	1.97 (5.32)	*T = 1.013*
**female = 40**	33.63 (63.44)	*P = 0.876*	0.80 (0.49)	*P = 0.353*	0.96 (1.12)	*P = 0.306*	1.07 (1.39)	*P = 0.314*

a Mean (SD).

### 3.2 Plasma miRNA epigenetic profiles

**Fig. 1:**
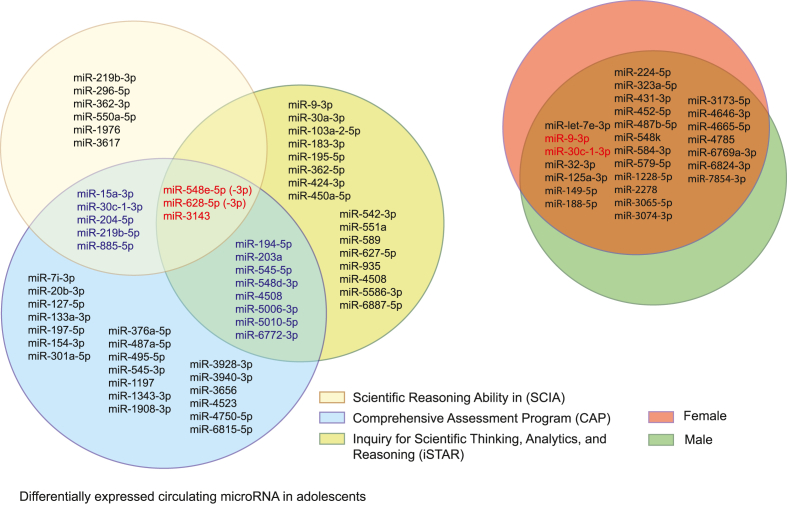
Differential expressed miRNAs assessed by th**ree tests.** This figure depicts a Venn diagram illustrating the overlap of differentially expressed miRNAs identified by three independent tests. The circles represent the sets of differentially expressed miRNAs identified by each test. Scientific Reasoning Ability in (SCIA) (light yellow); Comprehensive Assessment program (light blue) and iSTAR (yellow green); Male (Green) and Female (Red). (For interpretation of the references to colour in this figure legend, the reader is referred to the Web version of this article.)

In this study, we examined the relationship between miRNAs and their target messenger RNAs (mRNAs), which may influence learning and memory, and how this complex regulatory process varies based on the results of scientific thinking and reasoning tests. Plasma miRNA expression levels and their corresponding pathways were determined using different feature selection methods (test evaluation). We hypothesized that miRNAs are involved in the epigenetic mechanisms underlying the modulation of gene expression for behavior and memory maintenance. Consequently, 38 miRNAs with significant differential expression were identified between students with high and low academic performance. As shown in Fig. 1, 27 miRNAs exhibited substantial differential expression with high and low iSTAR scores, and 13 miRNAs exhibited significant differential expression with high and low scientific reasoning scores in the SCIA (Figs. 1), 27 miRNAs were identified as significantly different between genders, but only miR-30c-1-3p and miR-9-3p were two significantly important miRNAs in the gender group and the other groups. The research focus problem is beyond the gender dimension, but it will be deeply involved. There are only two sex forms in the world, but multiple gender variants, while the reproduction-related behavior of the gamete producers displays a much greater variability than just two prominent forms. The possible reason is that still a poorly understood cognitive memory system. Therefore, the current study will aim to control for the influence of gender on the effect of miRNA epigenetic profile on cognitive ability using statistics, prevent gender inequalities in required volunteers, and design the experiments.

### 3.3 Gene expression and miRNA levels were associated with cognitive abilities

As shown in Table 3, we identified a link between plasma biomarkers (gene expression levels) and academic performance (CAP) and cognitive ability (iSTAR). Consistent with our hypothesis that CaMKIIα is a major signaling molecule involved in both LTP and LTD, significant differences were found in the plasma levels of CaMKIIα in students with different CAP scores and iSTAR scores, with higher levels associated with higher scores. Thus, plasma CaMKIIα levels (ELISA, pg/mL) were positively and significantly linked with academic performance (CAP) and cognitive ability (iSTAR). In addition, the mRNA expression levels of plasma SIRT1 were negatively and significantly associated with cognitive ability (iSTAR). As presented in Table 3, the mRNA expression levels of plasma CaMKIIα and SIRT1 were positively and negatively, respectively, associated with cognitive ability (iSTAR) among Taiwanese adolescent students.

As shown in Table 4, a significant difference was only observed in the relationship between miRNA expression levels of miRNA-30c-1-3p and academic performance (CAP test) (*P* < 0.05). Validation of the miRNA epigenetic profile (Fig. 1) revealed a significant difference between academic performance (CAP test, *P* = 0.022) for miRNA-30c-1-3p. Pearson's correlation analysis revealed a correlation between different mRNAs and the expression levels of miRNAs, specifically revealing a two-tailed significant correlation between miRNA-30c-1-3p and miRNA-204–5p (Supplementary Table 3). Pearson's correlation analysis of the mRNA expression of MeCP2 also revealed a significant correlation between the mRNA expression levels of BDNF and HDAC9 (Supplementary Table 3). The null hypothesis was supported by the results for only BDNF and miRNA-204–5p in the paired-samples *t*-test (*P* = 0.492, Supplementary Table 4).

Overall, our findings indicate that miR-30c-1-3p may play a key role in both academic performance and cognitive ability. Its expression levels also appear to be correlated with other miRNAs and mRNAs. This study provides valuable insights into a potential biomarker of academic performance and cognitive ability, thereby warranting further investigation. In this study, we used a cellular model to examine the epigenetic mechanism underlying miRNA expression and to determine how synaptic plasticity initiates activation in induced LTP.

### 3.4 In cellular vitro study: CaMKIIα upregulation induces an autophosphorylation signaling cascade

**Fig. 2:**
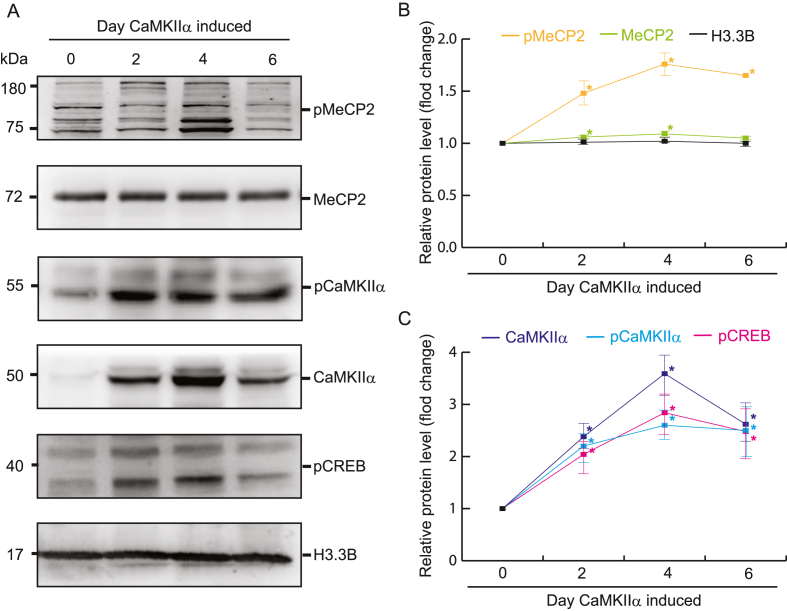
Phosphorylation of pMeCP2 and pCREB is consistent with the expression of pCaM**KIIα.** Total soluble protein from HEK293-derived cells were harvested at 2, 4, and 6 d after the induction of CaMKIIα expression with Dox. (**a**) Representative immunoblots displaying CaMKIIα, phosphor-CaMKIIα(T286), pCREB, MeCP2 and phosphs-MeCP2. H3.3 B was considered a loading control. (**b**, **c**) Quantification of relative protein expression levels. Data are presented as mean ± SD values. ∗P < 0.05, n = 5, Student's *t*-test.

**Fig. 3:**
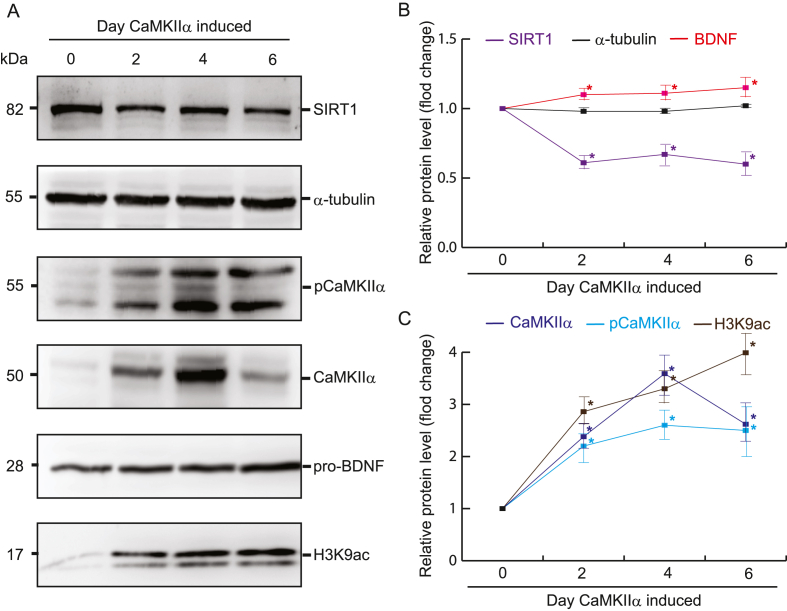
CaMKIIα phosphorylation increasing the acetylation of H3K9ac and inhibiting the expression of SIRT1. Total soluble protein from HEK293-derived cells were harvested at 2, 4, and 6 d after the induction of CaMKIIα expression with Dox. (**a**) Representative immunoblots displaying CaMKIIα, phosphor-CaMKIIα(T286), SIRT1, BDNF, and H3K9ac expression. α-Tubulin was considered loading control. (**b**, **c**) Quantification of relative protein expression levels. Data are presented as mean ± SD values. ∗P < 0.05, n = 5, Student's *t*-test.

To determine the effect of CaMKIIα upregulation in cellular vitro study, quantitative immunoblotting was performed using a stable human-derived HEK293 cell line because the author established this cell line and is typically used to examine relationships between epigenetic biomarkers and academic performance and neuronal disorders associated with neurocognitive disorders (Lee et al., 2021). The results indicated that CaMKIIα autophosphorylation proportionately increased with its expression; CaMKIIα expression was upregulated for 6 days, with CaMKIIα autophosphorylation plateauing after 2 days; this was followed by its slight downregulation after day 6. After the phosphorylation of CaMKIIα induced synaptic plasticity, a synchronous increase was observed in the levels of phosphorylated MeCP2 and CREB with the upregulation of CaMKIIα expression (Fig. 2). Simultaneously, CaMKIIα phosphorylation led to an increase in the acetylation of H3K9ac and the inhibition of SIRT1 expression (Fig. 3).

**Fig. 4:**
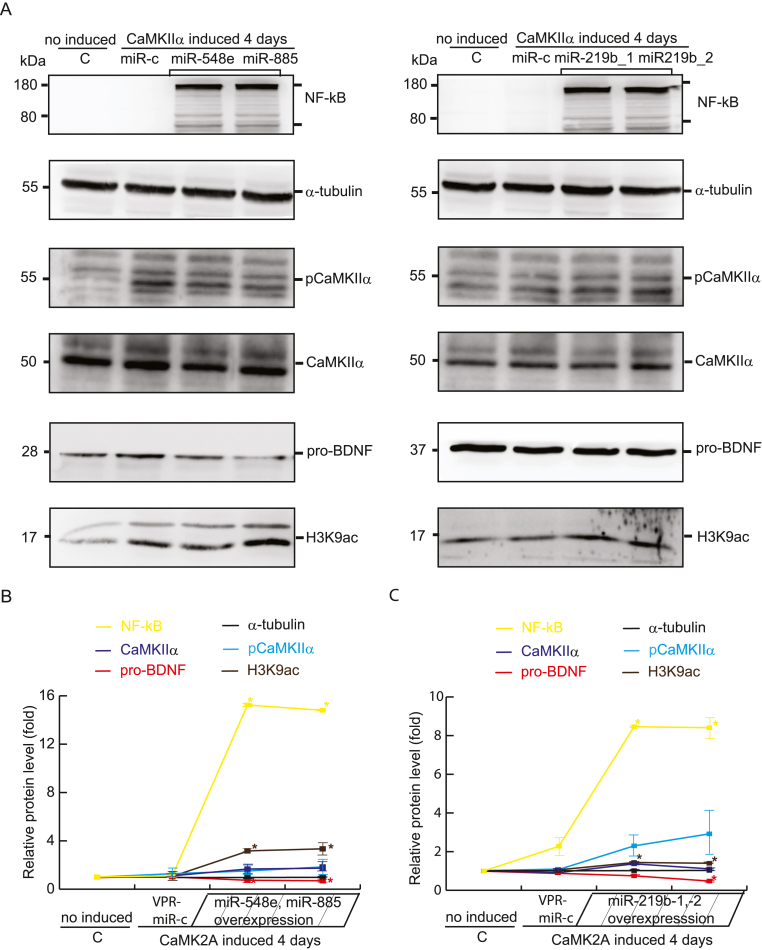
Overexpression of miRNAs inducing pro-inflammatory NF-κB and acetyl**ated H3K9.** (**a**) Representative immunoblotting showing the expression of CaMKIIα, and pCaMKIIα, in HEK293 cells transfected with the miR-548e, miR-885, and miRNA-291b-1, and -2) 4 days after the induction of CaMKIIα by Dox. Total soluble protein from HEK293-derived cells was harvested at day 6; (**b**, **c**) The relative protein levels were normalized to α-tubulin protein levels. The graph represents the average of three independent experiments with error bars indicating standard deviation (SD; mean ± SD). Statistical significance was determined using Student's *t*-test (∗*p* < 0.05). The predicated binding sites for miR-548e/885 and miR-219 b within the 3′ UTR of CaMKIIα were identified using TargetScan.

As shown in Fig. 4, overexpression of miR-548e/-885 and miR-219 b-1/-2 did not lead to the significant downregulation of the expression of CaMKIIα in human-derived HEK293 cells. Although the mechanisms underlying the expression of miRNAs in this synaptic signaling pathway remain unclear, we identified the multiple homeostatic regulation of MeCP2 and CaMKIIα as a potential mechanism (data not yet published). In this human-derived HEK293 cellular model, we discovered that the overexpression of miR-548e/885 and miR-219 b-1/-2 induced NF-κB immune inflammation and H3K9ac acetylation (Fig. 4).

## 4 Discussion

In individuals with impaired cognition, such as older individuals and individuals with dementia, Alzheimer's disease, brain trauma, and epilepsy, neurodegeneration is regarded to result from the disruption of multiple signaling pathways (Bahlakeh et al., 2021; Fehlmann et al., 2020). Research indicates that miRNAs contribute to many cellular and biological processes, such as cell growth, apoptosis, and differentiation (Mo, 2012). In patients with amnestic mild cognitive impairment, miRNAs play a neuroprotective role (Weinberg et al., 2015). Therefore, miRNAs are typically used as optimal, noninvasive, quantifiable biomarkers for disease diagnosis and prevention because of their stability in cells, tissues, and body fluids.

**Fig. 5:**
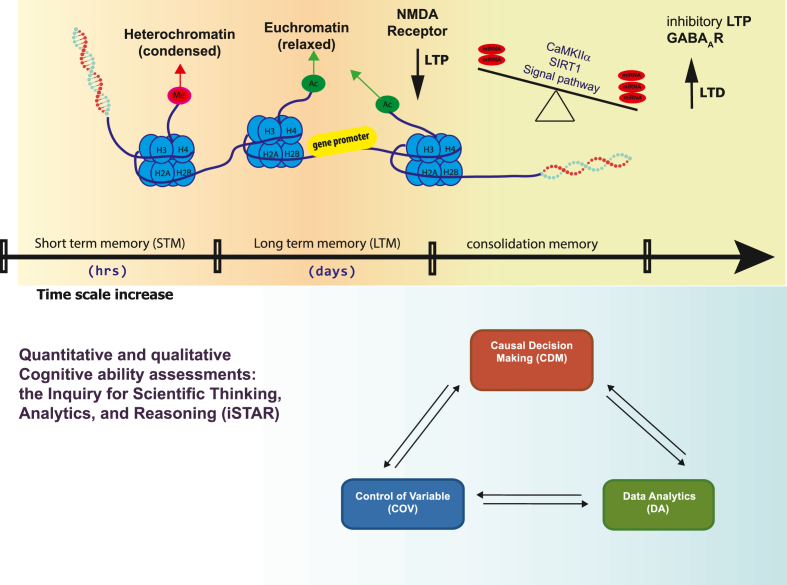
Research **framework.** The diagram depicts the role of Histone modifications in memory formation. Histone modifications, such as methylation and acetylation, lead to chromatin remodeling. In responding to calcium influx through synaptic NMDA receptors triggers long-term potentiation (LTP). Acetylated histones loosen the promoters of genes crucial for memory formation, making it more accessible for transcription machinery and increase genes expression. MicroRNAs that target CaMKIIα and SIRT1 are likely to be involved in the interplay between LTP and LTD for memory consolidation by affecting the balance of the processes. Quantitative and qualitative cognitive ability assessments will be supported by iSTAR test, which emphasize three areas of core skills that support scientific inquiry, including control of variables (COV), data analytics (DA), and causal decision making (CDM).

Cognitive processes generate new knowledge through acquiring, processing, storing, and retrieving information throughout the brain. These processes are primarily associated with presynaptic and postsynaptic plasticity and calcium-mediated signaling. In this scenario, the CaMKIIα/SIRT1/BDNF cascade plays a key role and interplay between inflammation and neural plasticity (Golia et al., 2019). Fig. 5 shows our research framework. To be transformed into long-term memory, short-term memory requires a temporal change in synaptic proteins and gene expression. Through precise mechanisms, genes are expressed into mRNAs and translated into proteins. According to recent studies, miRNAs serve as regulators of memory formation and cognitive function (Saab and Mansuy, 2014; Woldemichael and Mansuy, 2016).

Our investigation explored the potential influence of gender on cognitive abilities. We examined the regulation correlation between plasma biomarkers (gene expression levels) and both academic performance (CAP) and cognitive ability (iSTAR). Interestingly, positive regulation of CaMKIIα did not differ between genders. However, negative regulation of SIRT1 showed a significant gender-based disparity. This finding suggests that CaMKIIα might act as an initial regulatory point, influencing SIRT1 which is then modulated by gender-specific mechanisms. Additionally, analysis of microRNA profiles in human blood plasma (Fig. 1), miR-30c-1-3p and miR-9-3p were only two significant differences both in the gender group and the other groups. This specific microRNA has been previously linked to sex hormone function (Chen et al., 2019; Huang et al., 2022; Vachirayonstien and Yan, 2016).

In this study, we developed an epigenetic memory model to examine the epigenetic mechanism underlying the expression of miRNAs and to determine how synaptic plasticity induces LTP. We also investigated how molecular biology can be used to further understand the relationships between cognitive ability and academic performance in adolescents.

**Fig. 6:**
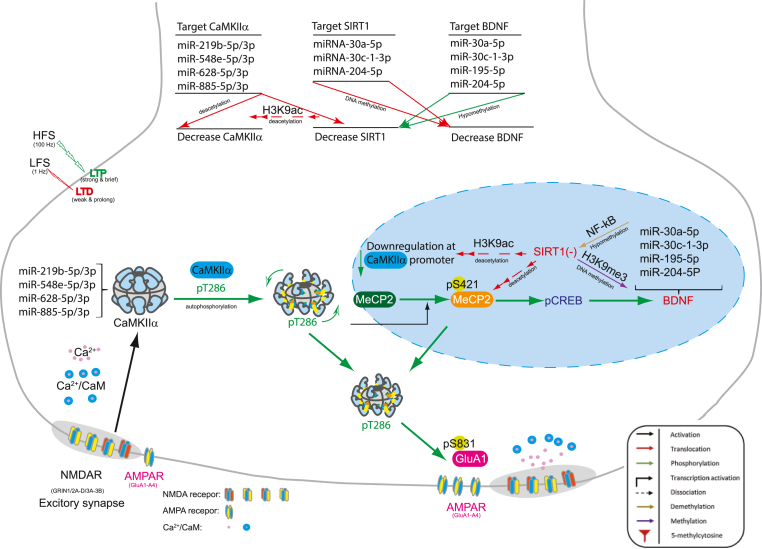
A plausible role of microRNAs, NF-kB and SIRT1 in regulating synaptic plasticity by targeting SIRT1, CaMKIIα, and BDNF. Neuronal activity leads to an influx of calcium (Ca^2+^) ions into the postsynaptic neuron. Ca^2+^ binds to calmodulin (CaM), which in turn activates CaMKIIα by inducing a conformational change. This CaM-Ca^2+^-dependent activation allows CaMKIIα to autophosphorylate at a threonine-286), creating an autonomous and persisted CaM-independent activity state which actives GluA1 at excitatory synapses and MeCP2-CREB-BDNF signaling cascades. The negative regulation under hypomethylation conditions, we identified hypomethylation and elevated NF-κB activity, decreased SIRT1, and downregulation of CaMKIIα expression. We postulate that CaMKIIα-mediated synaptic plasticity can be modulated by the synergistic action of miRNAs (particularly miR-30a/30c-1/195/204), NF-kB and SiRT1.

In a previous study (Lee et al., 2019), we screened the genetic and epigenetic factors associated with the cognitive ability of adolescents, and we discovered that multiple epigenetic biomarkers regulating MeCP2 homeostasis were associated with students' academic performance. In another study (Lee et al., 2021), we discovered that epigenetic regulation related to N-methyl-D-aspartate (NMDA) receptors contributed to reasoning ability, with elevated CaMKIIα levels upregulating the expression of pCaMKIIα and GluA1. We also discovered that MeCP2, a downstream target of CaMKIIα, upregulated the expression of CaMKIIα, indicating that CaMKIIα and MeCP2 formed an autoregulatory signal transduction loop with positive feedback. We speculated that changes in the expression levels of CaMKIIα affected the cognitive ability of adolescents. In this study, we discovered that miRNAs' profile was associated with adolescents' cognitive ability. Therefore, we hypothesized that plasma miRNAs can modulate the CaMKIIα/SIRT1 signaling pathway as a biomarker of cognitive ability in adolescents (Fig. 6).

Overall, our cell model of synaptic initiation revealed three key findings. First, CaMKIIα functions as a gateway for epigenetic memory formation. In this study, we developed a model in which activating the CaMKIIα signaling pathway directly induces the formation of epigenetic memory, which is consistent with the models in previous studies (Lee et al., 2019, 2021). Further detailed studies are required to identify the complex relationship between CaMKIIα and homeostatic regulators such as MeCP2. Second, SIRT1 has an inhibitory effect on CaMKIIα. In this study, we discovered that SIRT1 functions as a negative regulator of CaMKIIα activity, thereby hindering signaling and memory formation. This finding adds another dimension to the complex mechanism of synaptic initiation. Third, miRNAs and SIRT1 exhibit synergy. Under reduced methylation conditions (hypomethylation, as depicted in Fig. 4, with increased levels of NF-κB), certain miRNAs (e.g., miR-30a-3p, miR-30c-1-3p, miR-195–5p, and miR-204–5p) cooperate with SIRT1 to amplify its negative regulation of CaMKIIα, further fine-tuning the synaptic signaling process.

Current research indicates a complex relationship between genetic and epigenetic factors, which is influenced by the environment, affects the vulnerability of humans to stressors, shapes the evolution of cognitive decline with aging, and even lays the foundation for potential cellular damage (Barger, 2016; Barter and Foster, 2018; Fehlmann et al., 2020; Islam et al., 2021). Certain miRNAs have been linked to crucial brain functions by targeting essential proteins such as CaMKIIα (Gupta et al., 2020, Jin et al., 2022, Wang et al., 2018), SIRT1 (Shen et al., 2018, Shen et al., 2019, Zhou et al., 2020), and CREB (Xiang et al., 2016, Xiang et al., 2022).

In this study, we examined the complex interplay between epigenetic modifications, miRNA expression, and signaling pathways, shaping the cognitive function of adolescents. We identified hypomethylation and elevated NF-κB activity as key drivers of synaptic plasticity, influencing learning and memory formation (Fig. 6). We also uncovered the synergistic role of certain miRNA families, particularly miR-30 and miR-204, in conjunction with SIRT1 protein in fine-tuning this process (Fig. 6). Overall, our findings indicated a link between readily measurable plasma miRNA levels and cognitive abilities evaluated using standardized tests (CAP and iSTAR). These findings lay the foundation for exploring these miRNAs as potential biomarkers of cognitive function in adolescents. To delve deeper into the underlying mechanisms, we conducted comprehensive experiments of gene and miRNA expression patterns in cellular models (Fig. 2, Fig. 3, Fig. 4). These experiments revealed complex correlations between the levels of mRNA and miRNA (Supplementary Table 3), further substantiating the proposed interplay between miRNAs and CaMKIIα in regulating synaptic plasticity under hypomethylation conditions (Fig. 6). Given our findings, we proposed a novel hypothesis involving the synergistic action of miRNAs in modulating CaMKIIα-mediated synaptic plasticity under hypomethylation conditions. This framework provides insights into the complex molecular aspects of cognitive function in adolescents. Generally, hypomethylation and increased NF-κB levels play a key role in determining the direction of synaptic plasticity (Yakovleva et al., 2011; Yan et al., 2016; Ye et al., 2019). In addition, the synergy between miRNAs and SIRT1 involves miRNA-30 (Cattaneo et al., 2020, Kumar and Li, 2022, Tan et al., 2020, Vachirayonstien and Yan, 2016) and miR-204–5p (Xiang et al., 2016, Xiang et al., 2022). It also indicates that specific plasma miRNAs, particularly miR-30a/30c-1/195/204, are potential biomarkers for cognitive assessment, thereby laying the foundation for further personalized approaches aimed at supporting cognitive development in young individuals.

Overall, our findings revealed how epigenetic modifications, miRNA expression, and signaling pathways collaboratively affect cognitive function in adolescents. The CaMKII/SIRT1 signaling pathway is an initiator of epigenetic memory and has high predictive power for students’ cognitive abilities and academic performance. In addition, hypomethylation and increased NF-κB activity as key components of synaptic plasticity, influencing learning and memory (Yakovleva et al., 2011). Certain families of miRNAs, such as miR-30 and miR-204, work in synergy with SIRT1, thereby fine-tuning the cognitive process. Overall, this study bridges the gap between the molecular machinery within cells and the readily measurable variables in the plasma of adolescents. Plasma miRNA levels, particularly those of miR-30a/30c-1/195/204, are regarded as promising biomarkers for cognitive assessment, providing an overall view of variations in learning and memory.

### 4.1 Limitation

This study has several limitations that warrant consideration. While the participants were of similar ages and educational backgrounds, we acknowledge that sex alone cannot fully explain observed cognitive differences. Human cognition is a complex interplay of numerous factors, including environment, culture, and individual experiences. Due to the inability to quantitatively assess these factors in the present study, we refrain from over-interpreting the results or speculating on specific causal mechanisms. The inherent variability and complexity of learning behaviors further complicate the establishment of definitive conclusions. Specifically, the absence of a pre-study assessment of relevant variables at the individual or meta-level limits our ability to draw strong connections between the factors examined. Future research should incorporate rigorous hypothesis testing, including collecting and analyzing data on students’ academic performance, cognitive abilities, behavior, and relevant genetic and environmental information.

## CRediT authorship contribution statement

**Li-Ching Lee:** Writing – original draft, Investigation, Data curation, Conceptualization. **Ming-Tsan Su:** Writing – original draft, Investigation, Data curation, Conceptualization. **Lei Bao:** Writing – review & editing, Visualization. **Po-Lei Lee:** Visualization. **Shane Tutwiler:** Visualization, Validation. **Ting-Kuang Yeh:** Writing – review & editing, Supervision. **Chun-Yen Chang:** Writing – review & editing, Supervision.

## Declaration of competing interest

**Teaser:** MicroRNAs could be the key to unlocking adolescent cognitive potential.

## Data Availability

The data that has been used is confidential.
