# Supplementary material for: MicroRNAs modulate CaMKIIα/SIRT1 signaling pathway as a biomarker of cognitive ability in adolescents
Source: Brain Behav Immun Health. 2025 Feb 24;44:100970. doi: 10.1016/j.bbih.2025.100970 (PMC11919301; doi:10.1016/j.bbih.2025.100970)
Supplement: Multimedia component 1 [file mmc1.docx]

**Supplementary Materials for**

**MicroRNAs modulate CaMKIIα/SIRT1 signaling pathway as a biomarker of cognitive ability in adolescents**

Li-Ching Lee *et al.*

*Corresponding author. Email: [tkyeh@ntnu.edu.tw](mailto:tkyeh@ntnu.edu.tw); changcy@ntnu.edu.tw

**This PDF file includes:**

Supplementary Text

Tables S1 to S3

Extended Data Excel file 1 to 4

**Other Supplementary Materials for this manuscript include the following:**

Extended data for Fig. 1 (Extended data Excel file 1-4 for human plasma miRNA profiling)

**Supplementary text**

**Human plasma miRNA profiling**

Whole blood samples were collected in tubes containing ethylenediaminetetraacetic acid (EDTA). Plasma was obtained by centrifuging at 2000 ×*g* for 15 min at 4℃, and stored at −80℃ until miRNA extraction and sequencing. miRNA was extracted from 40 pre-screened samples using the miRNeasy Serum/Plasma Kit (Qiagen, Hilden, Germany). Small RNA libraries were prepared using Ion Total RNA-Seq Kit v2 (Life Technologies, Carlsbad, CA, USA). Following library preparation, samples were pooled in an equal molar ratio for template bead preparation. Subsequently, sequencing beads were prepared and enriched according to the manufacturer’s instructions (Ion Chef, Next-Generation Sequencing). Sequencing was performed using the Ion 540 chip on the Ion GeneStudio S5 Prime platform (Thermo Fisher Scientific, Waltham, MA, USA) with two sequencing runs per initialization, following the instructions. Sequences were aligned to the human reference genome (hg19) and base calling were performed using built-in Torrent Suite Software v5.16.1 (Thermo Fisher Scientific, Waltham, MA, USA). Differential expression analysis of miRNAs was conducted using Partek Genomics Suite v7.0 (Partek Inc., St. Louis, MO, USA).

**Extended data Fig. 1 (Extended data Excel file 1-4 for human plasma miRNA profiling)**

**Extended data 1.** Fxcel.data MicroRNAs (miRNAs) for differentially expressed in association with the CAP test. To investigate the potential association between microRNA (miRNA) expression and academic performance, miRNA profiling was performed on plasma samples collected from a cohort of students. A performance rating higher than 85 was designated as the higher academic performance group, whereas a performance rating lower than 60 was designated as the lower academic performance group.

**Extended data 2.** Fxcel data for miRNAs differentially expressed in association with the iSTAR assessment. Plasma miRNA expression profiles from a cohort of 40 students were compared based on the logits of the iSTAR assessment. Those iSTAR scoring >= 9 designated as the higher performance group (n=20), while those scoring < 9 were designated as the lower performance group (n=20).

**Extended data 3.** Excel data for miRNA differentially expressed in association with the scientific reasoning subset score in the situation-based career interest assessment (SCIA). Plasma miRNA expression profiles from a cohort of 40 students were compared based on the scientific reasoning score in SCIA. A score higher than 108 was designated as the higher scientific reasoning group (n=20), whereas a score lower than 108 was designated as the lower scientific reasoning group (n=20) (Total score was 140).

**Extended data 4.** Excel data for miRNA differentially expressed in association with gender. This study investigated potential sex-based differences in microRNA (miRNA) expression profiles. Plasma samples were collected from 40 participants (20 males and 20 females). Plasma miRNA expression profiles were compared based on gender.

**Supplementary data Table 1** The sequencing coverage of Human plasma miRNA profiling

**Supplementary data Table 2** Pearson’s correlation analysis results of academic performance (CAP test) and cognitive ability (iSTAR).

**Supplementary data Table 3** Pearson’s correlation analysis results of different mRNAs and miRNA expression levels.

**Supplementary data Table 4** Gene (mRNA) and miRNA expression levels in a paired-samples *t* test

**Supplementary data Table 5** In vitro cellular model study, one-way analysis of variance (ANOVA), and a post hoc least significant difference test

**Table S1 | The sequencing coverage of Human plasma miRNA**

| **Sample** | **Total Bases** | **Reads** | **Mean Read Length** |
| --- | --- | --- | --- |
| 110007 | 12,452,707 | 523,141 | 23 bp |
| 110010 | 21,032,143 | 830,953 | 25 bp |
| 110012 | 7,853,680 | 404,779 | 19 bp |
| 110013 | 12,871,713 | 515,003 | 24 bp |
| 110019 | 19,973,953 | 821,728 | 24 bp |
| 110028 | 19,783,561 | 789,824 | 25 bp |
| 110032 | 27,783,300 | 1,063,873 | 26 bp |
| 110035 | 8,047,128 | 376,806 | 21 bp |
| 110042 | 17,372,118 | 765,697 | 22 bp |
| 110045 | 31,734,967 | 1,117,042 | 28 bp |
| 110046 | 7,576,027 | 323,866 | 23 bp |
| 110047 | 25,244,261 | 933,597 | 27 bp |
| 110049 | 6,355,720 | 248,439 | 25 bp |
| 110055 | 10,263,224 | 469,552 | 21 bp |
| 110062 | 25,018,670 | 991,515 | 25 bp |
| 110063 | 5,417,472 | 230,176 | 23 bp |
| 110064 | 13,056,330 | 629,642 | 20 bp |
| 110066 | 9,741,510 | 395,791 | 24 bp |
| 110075 | 5,922,028 | 276,891 | 21 bp |
| 110077 | 23,636,181 | 1,039,497 | 22 bp |
| 110082 | 4,874,816 | 200,585 | 24 bp |
| 110088 | 25,173,595 | 1,011,988 | 24 bp |
| 110091 | 3,302,988 | 144,961 | 22 bp |
| 110093 | 27,130,438 | 1,010,102 | 26 bp |
| 110098 | 4,630,661 | 202,897 | 22 bp |
| 110100 | 12,828,410 | 624,117 | 20 bp |
| 110101 | 19,234,717 | 785,591 | 24 bp |
| 110107 | 11,636,956 | 500,627 | 23 bp |
| 110111 | 3,181,976 | 137,758 | 23 bp |
| 110123 | 6,719,654 | 302,210 | 22 bp |
| 110125 | 4,657,633 | 227,448 | 20 bp |
| 110132 | 5,319,138 | 233,116 | 22 bp |
| 110145 | 2,977,290 | 135,360 | 21 bp |
| 110163 | 9,724,931 | 423,213 | 22 bp |
| 110173 | 10,723,245 | 495,599 | 21 bp |
| 110190 | 13,458,820 | 584,295 | 23 bp |
| 110196 | 5,319,138 | 196,891 | 21 bp |
| 110212 | 2,606,098 | 117,627 | 22 bp |
| 110218 | 24,529,390 | 1,109,586 | 22 bp |
| 110226 | 3,288,045 | 148,118 | 22 bp |

**Table S2 | Pearson’s correlation analysis results of academic performance (CAP test) and cognitive ability (iSTAR).**

|  | | **Comprehensive Assessment Program (CAP)** | **iSTAR test** | **Control of variables** | | **Data analytics** | **Causal decision making** |
| --- | --- | --- | --- | --- | --- | --- | --- |
| **Comprehensive Assessment Program**  **(CAP)** | Pearson correlation | 1 | .718^**^ | .640^**^ | .401^**^ | | .512^**^ |
|  | Significance（Two-tailed） |  | .000 | .000 | .000 | | .000 |
|  | N | 78 | 78 | 78 | 78 | | 78 |
| **iSTAR** | Pearson correlation | .718^**^ | 1 | .788^**^ | .659^**^ | | .734^**^ |
|  | Significance（Two-tailed） | .000 |  | .000 | .000 | | .000 |
|  | N | 78 | 78 | 78 | 78 | | 78 |
| **Control of variables** | Pearson correlation | .640^**^ | .788^**^ | 1 | .250^*^ | | .409^**^ |
|  | Significance（Two-tailed） | .000 | .000 |  | .027 | | .000 |
|  | N | 78 | 78 | 78 | 78 | | 78 |
| **Data analytics** | Pearson correlation | .401^**^ | .659^**^ | .250^*^ | 1 | | .224^*^ |
|  | Significance（Two-tailed） | .000 | .000 | .027 |  | | .049 |
|  | N | 78 | 78 | 78 | 78 | | 78 |
| **Causal decision making** | Pearson correlation | .512^**^ | .734^**^ | .409^**^ | .224^*^ | | 1 |
|  | Significance（Two-tailed） | .000 | .000 | .000 | .049 | |  |
|  | N | 78 | 78 | 78 | 78 | | 78 |

^**^Correlations significant at the 0.01 level (two-tailed). ^*^Correlations significant at the 0.05 level (two-tailed).

**Table S3 | Pearson’s correlation analysis results of different mRNAs and miRNA expression levels.**

|  | | **MeCP2** | **BDNF** | **SIRT1** | **HDAC9** | **ELSIA CaMKIIα** | **miR-30a-5p** | **miR-30c-1-3p** | **miR195-5p** | **miR-204-5p** | |  |
| --- | --- | --- | --- | --- | --- | --- | --- | --- | --- | --- | --- | --- |
| **MeCP2** | Pearson correlation | 1 | .555^**^ | .063 | .581^**^ | -.215 | .100 | -.142 | -.015 | | -.018 | |
|  | Significance (Two tailed) |  | .000 | .586 | .000 | .059 | .384 | .215 | .896 | | .874 | |
|  | N | 78 | 78 | 78 | 78 | 78 | 78 | 78 | 78 | | 78 | |
| **BDNF** | Pearson correlation | .555^**^ | 1 | .148 | .156 | -.176 | .087 | .025 | -.038 | | .014 | |
|  | Significance (Two tailed) | .000 |  | .195 | .173 | .122 | .450 | .828 | .741 | | .907 | |
|  | N | 78 | 78 | 78 | 78 | 78 | 78 | 78 | 78 | | 78 | |
| **SIRT1** | Pearson correlation | .063 | .148 | 1 | .308^**^ | -.186 | -.154 | .075 | .092 | | .091 | |
|  | Significance (Two tailed) | .586 | .195 |  | .006 | .104 | .179 | .517 | .423 | | .430 | |
|  | N | 78 | 78 | 78 | 78 | 78 | 78 | 78 | 78 | | 78 | |
| **HDAC9** | Pearson correlation | .581^**^ | .156 | .308^**^ | 1 | -.204 | -.092 | -.105 | .136 | | -.043 | |
|  | Significance (Two tailed) | .000 | .173 | .006 |  | .073 | .424 | .361 | .236 | | .708 | |
|  | N | 78 | 78 | 78 | 78 | 78 | 78 | 78 | 78 | | 78 | |
| **ELSIA CaMKIIα** | Pearson correlation | -.215 | -.176 | -.186 | -.204 | 1 | -.096 | .109 | -.073 | | -.058 | |
|  | Significance (Two tailed) | .059 | .122 | .104 | .073 |  | .403 | .343 | .523 | | .614 | |
|  | N | 78 | 78 | 78 | 78 | 78 | 78 | 78 | 78 | | 78 | |
| **miR-30a-5p** | Pearson correlation | .100 | .087 | -.154 | -.092 | -.096 | 1 | -.095 | .003 | | -.058 | |
|  | Significance (Two tailed) | .384 | .450 | .179 | .424 | .403 |  | .407 | .976 | | .617 | |
|  | N | 78 | 78 | 78 | 78 | 78 | 78 | 78 | 78 | | 78 | |
| **miR-30c-1-3p** | Pearson correlation | -.142 | .025 | .075 | -.105 | .109 | -.095 | 1 | .057 | | .491^**^ | |
|  | Significance (Two tailed) | .215 | .828 | .517 | .361 | .343 | .407 |  | .617 | | .000 | |
|  | N | 78 | 78 | 78 | 78 | 78 | 78 | 78 | 78 | | 78 | |
| **miR-195-5p** | Pearson correlation | -.015 | -.038 | .092 | .136 | -.073 | .003 | .057 | 1 | | -.033 | |
|  | Significance (Two tailed) | .896 | .741 | .423 | .236 | .523 | .976 | .617 |  | | .777 | |
|  | N | 78 | 78 | 78 | 78 | 78 | 78 | 78 | 78 | | 78 | |
| **miR-204-5p** | Pearson correlation | -.018 | .014 | .091 | -.043 | -.058 | -.058 | .491^**^ | -.033 | | 1 | |
|  | Significance (Two tailed) | .874 | .907 | .430 | .708 | .614 | .617 | .000 | .777 | |  | |
|  | N | 78 | 78 | 78 | 78 | 78 | 78 | 78 | 78 | | 78 | |

^**^Correlations significant at the 0.01 level (two-tailed). ^*^Correlations significant at the 0.05 level (two-tailed).

**Table S4 | Gene (mRNA) and miRNA expression levels in a paired-samples *t* test**

| **Paired sample T test gene expression level (mRNA)** | **miRNA expression level** | **Mean (M)** | **Standard Deviation (SD)** | **Standard error of mean (SEM)** | | ***t-statistic value*** | **Significant (two tail)** |
| --- | --- | --- | --- | --- | --- | --- | --- |
| **MeCP2 gene expression level** | miR*-*30a*-*5p | -30.23 | 60.3 | | 6.82 | *T = -4.427* | *P = 0.000* |
| **N = 78, df = 77** | miR-30c-1-3p | 3.65 | 4.69 | | 0.53 | *T = 6.863* | *P = 0.000* |
|  | miR-195-5p | 3.65 | 4.67 | | 0.53 | *T = 6.907* | *P = 0.000* |
|  | miR-204-5p | 2.98 | 6.09 | | 0.69 | *T = 4.32* | *P = 0.000* |
| **SIRT1 gene expression level** | miR-30a*-*5p | -34.38 | 60.62 | | 6.86 | *T = -5.009* | *P = 0.000* |
| **N = 78 df = 77** | miR-30c-1-3p | -0.51 | 0.61 | | 0.07 | *T = -7.263* | *P = 0.000* |
|  | miR-195-5p | -0.5 | 0.88 | | 0.1 | *T = -5.029* | *P = 0.000* |
|  | miR-204-5p | -1.17 | 3.92 | | 0.44 | *T = -2.648* | *P = 0.000* |
| **BDNF gene expression level** | miR-30a*-*5p | -32.87 | 60.46 | | 6.84 | *T = -7.263* | *P = 0.000* |
| **N = 78, df = 77** | miR-30c-1-3p | 1.01 | 1.84 | | 0.21 | *T = 4.829* | *P = 0.000* |
|  | miR-195-5p | 1.01 | 1.99 | | 0.23 | *T = 4.481* | *P = 0.000* |
|  | miR-204-5p | 0.36 | 4.29 | | 0.49 | *T = 0.691* | ***P = 0.492*** |
| **HDAC9 gene expression level** | miR-30a-5p | -32.75 | 60.75 | | 3.88 | *T = -4.761* | *P = 0.000* |
| **N = 78, df = 77** | miR-30c-1-3p | 1.13 | 1.7 | | 0.19 | *T = 5.876* | *P = 0.000* |
|  | miR-195-5p | 1.13 | 1.66 | | 0.19 | *T = 6.044* | *P = 0.000* |
|  | miR-204-5p | 0.46 | 4.28 | | 0.48 | *T = 0.946* | *P = 0.000* |
| **ELISA CaMKIIα expression level** | miR-30a-5p | 415.06 | 236.71 | | 26.8 | *T = 15.486* | *P = 0.000* |
| **N = 78, df = 77** | miR-30c-1-3p | 448.94 | 223.02 | | 25.25 | *T = 17.778* | *P = 0.000* |
|  | miR-195-5p | 448.94 | 223.15 | | 25.27 | *T = 17.768* | *P = 0.000* |
|  | miR-204-5p | 448.27 | 223.34 | | 25.29 | *T = 17.726* | *P = 0.000* |

**Cell culture and transfection**

HEK293 cells expressing either CaMKIIα or MeCP2 was generated using an inducible Flp-In T-REx system (Invitrogen, Carlsbad, CA, USA). Briefly, the HEK293 cells were cotransfected with a pOG44 plasmid (constitutively expressing Flp recombinase) and a pcDNA5/FRT/TO-MeCP2 plasmid, and the stably transfected cells were obtained as per the supplier’s instructions. HEK 293-derived Flp-In host cells, expressing either CaMKIIα or MeCP2, were generated as previously described (Lee et al., 2012). Cell lines were cultured in medium containing 5 mg/mL blasticidin and 100 mg/mL hygromycin. To induce the expression of either CaMKIIα or MeCP2, doxycycline was added to the medium at a final concentration of 1 mg/mL for 2-6 days. For transient overexpression, HEK293-drived CaMKIIα stable cells were plated into 6-well (on coverslips, 1x10^5^/ well) dishes, grown with doxycycline induced four days, and transient transfected with miRNA-548e, miR-885 and miR-219b (All-in-one / CRISPRa-VPR) from the National RNAi Core Facility at Academia Sinica in Taiwan. The cells were grown for 48hr for the following immunocytochemical staining.

**Western blotting**

HEK293- and SH-SY5Y-derived stable cells treated with 3 µM Calmodulin (CaM, Cat. No. C4874, Sigma-Aldrich, St. Louis, MO, USA) were lysed in a buffer containing 50 mM Tris-HCl, 150 mM NaCl, 1 mM EDTA, 1 mM ethylene glycol-bis(β-aminoethyl ether)-N,N,N′,N′-tetraacetic acid, 0.1% sodium dodecyl sulfate, 0.5% sodium deoxycholate, 1% Triton X-100, and a protease inhibitor cocktail (Life Technologies, Thermo Fisher Scientific) at designated time points (0, 2, 4, and 6 days). Total soluble protein was obtained by centrifugation at 15,000 ×*g* for 10 min at 4°C. Protein concentrations were determined using the Bradford assay (Cat. No. 5000002; Bio-Rad Laboratories Hercules, CA, USA). For each sample, a total of 25 µg proteins were separated using 12% sodium dodecyl sulfate–polyacrylamide gel electrophoresis (SDS-PAGE), and electroblotted onto nitrocellulose membranes. Protein blots were blocked with 10% nonfat milk. Antibodies used and their dilutions: anti-MeCP2 (1:2000; GeneTex, Irvine, CA, USA), anti-pMeCP2 (phospho-S80, 1:1000; GeneTex), anti-CaMKIIα (1:1000; GeneTex), anti-pCaMKIIα (phospho-T286, 1:1000; GeneTex), anti-BDNF (1:2000; GeneTex), anti-SIRT1 (1:1000; GeneTex), anti-H3K9ac (1:1000; GeneTex), anti-α-tubulin (1:1000; Merck Millipore), anti-NF-κB (1:1000; Cell Signaling Technology), and anti-H3.3B (1:2000; GeneTex). Horseradish peroxidase (HRP)-conjugated goat anti-mouse and goat anti-rabbit IgG antibodies (1:10,000 dilution; Jackson ImmunoResearch, West Grove, PA, USA) were used in conjunction with an ECL chemiluminescent substrate (Millipore, Burlington, MA, USA) for signal detection.

**Table S5 | In vitro cellular model study, one-way analysis of variance (ANOVA), and a post hoc least significant difference test**

| **Group** | | **N** | **Mean** | **SD** | **F** | ***p*** | **Post hoc**  **(Scheffe, Games-Howell)** |
| --- | --- | --- | --- | --- | --- | --- | --- |
| MeCP2 | Standard (0) | 7 | 1.00 | 0.00 | 2.686 | *.069* |  |
|  | 2 days (2) | 7 | 1.06 | 0.07 |  |  |  |
|  | 4 days (4) | 7 | 1.09 | 0.08 |  |  |  |
|  | 6 days (6) | 7 | 1.05 | 0.07 |  |  |  |
|  | total | 28 | 1.05 | 0.07 |  |  |  |
| pMeCP2 | Standard (0) | 7 | 1.00 | 0.00 | 4.660 | *.011* | 0<4 |
|  | 2 days (2) | 7 | 1.49 | 0.47 |  |  |  |
|  | 4 days (4) | 7 | 1.86 | 0.44 |  |  |  |
|  | 6 days (6) | 7 | 1.65 | 0.62 |  |  |  |
|  | total | 28 | 1.50 | 0.53 |  |  |  |
| BDNF | Standard (0) | 7 | 1.00 | 0.00 | 2.384 | *.094* |  |
|  | 2 days (2) | 7 | 1.10 | 0.10 |  |  |  |
|  | 4 days (4) | 7 | 1.11 | 0.12 |  |  |  |
|  | 6 days (6) | 7 | 1.15 | 0.17 |  |  |  |
|  | total | 28 | 1.09 | 0.12 |  |  |  |
| pCREB | Standard (0) | 7 | 1.00 | 0.00 | 7.929 | *.001* | 0<2  0<4  0<6 |
|  | 2 days (2) | 7 | 2.04 | 0.74 |  |  |  |
|  | 4 days (4) | 7 | 2.84 | 0.86 |  |  |  |
|  | 6 days (6) | 7 | 2.48 | 0.97 |  |  |  |
|  | total | 28 | 2.09 | 1.00 |  |  |  |
| CaMK2A | Standard (0) | 7 | 1.00 | 0.00 | 13.342 | *.000* | 0<2  0<4  2<4 |
|  | 2 days (2) | 7 | 2.38 | 0.59 |  |  |  |
|  | 4 days (4) | 7 | 3.59 | 0.75 |  |  |  |
|  | 6 days (6) | 7 | 2.37 | 1.20 |  |  |  |
|  | total | 28 | 2.33 | 1.18 |  |  |  |
| pCaMK2A | Standard (0) | 7 | 1.00 | 0.00 | 13.396 | *.000* | 0<2  0<4  0<6 |
|  | 2 days (2) | 7 | 2.20 | 0.38 |  |  |  |
|  | 4 days (4) | 7 | 2.60 | 0.58 |  |  |  |
|  | 6 days (6) | 7 | 2.50 | 0.81 |  |  |  |
|  | total | 28 | 2.07 | 0.82 |  |  |  |
| H3.3B (1) | Standard (0) | 7 | 1.00 | 0.00 | .384 | *.766* |  |
|  | 2 days (2) | 7 | 1.03 | 0.06 |  |  |  |
|  | 4 days (4) | 7 | 1.00 | 0.06 |  |  |  |
|  | 6 days (6) | 7 | 1.02 | 0.08 |  |  |  |
|  | total | 28 | 1.01 | 0.06 |  |  |  |
| H3.3B (2) | Standard (0) | 7 | 1.00 | 0.00 | .953 | *.431* |  |
|  | 2 days (2) | 7 | 1.01 | 0.03 |  |  |  |
|  | 4 days (4) | 7 | 1.02 | 0.03 |  |  |  |
|  | 6 days (6) | 7 | 1.00 | 0.03 |  |  |  |
|  | total | 28 | 1.01 | 0.03 |  |  |  |
| SIRT1 | Standard (0) | 6 | 1.00 | 0.00 | 16.438 | *.000* | 0>2  0>4  0>6 |
|  | 2 days (2) | 6 | 0.61 | 0.10 |  |  |  |
|  | 4 days (4) | 6 | 0.67 | 0.14 |  |  |  |
|  | 6 days (6) | 6 | 0.60 | 0.15 |  |  |  |
|  | total | 24 | 0.72 | 0.20 |  |  |  |
| H3.K9ac | Standard (0) | 3 | 1.00 | 0.00 | 12.191 | *.002* | 0<2  0<4  0<6 |
|  | 2 days (2) | 3 | 2.86 | 0.66 |  |  |  |
|  | 4 days (4) | 3 | 3.30 | 0.73 |  |  |  |
|  | 6 days (6) | 3 | 3.99 | 0.80 |  |  |  |
|  | total | 12 | 2.79 | 1.28 |  |  |  |
| a-tubulin | Standard (0) | 3 | 1.00 | 0.00 | 4.232 | *.046* |  |
|  | 2 days (2) | 3 | 0.98 | 0.03 |  |  |  |
|  | 4 days (4) | 3 | 0.97 | 0.03 |  |  |  |
|  | 6 days (6) | 3 | 1.02 | 0.02 |  |  |  |
|  | total | 12 | 0.99 | 0.03 |  |  |  |
